# NF-κB/STAT3/PI3K signaling crosstalk in iMyc^Eμ ^B lymphoma

**DOI:** 10.1186/1476-4598-9-97

**Published:** 2010-04-30

**Authors:** Seong-Su Han, Hwakyung Yun, Dong-Ju Son, Van S Tompkins, Liangping Peng, Seung-Tae Chung, Joong-Su Kim, Eun-Sung Park, Siegfried Janz

**Affiliations:** 1 University of Iowa Carver College of Medicine, Department of Pathology, Iowa City, IA, USA; 2 Hanseo University, Department of Biological Sciences, Choognam, South Korea; 3 University of Medicine and Dentistry of New Jersey, The Public Health Research Institute Center, Newark, NJ, USA; 4 National Institute of Toxicological Research, Korea Food and Dug Administration, Seoul, South Korea; 5 Korea Research Institutes of Bioscience and Biotechnology, Daejeon, South Korea; 6 Department of Systems Biology, University of Texas M. D. Anderson Cancer Center, Houston, TX, USA

## Abstract

**Background:**

Myc is a well known driver of lymphomagenesis, and Myc-activating chromosomal translocation is the recognized hallmark of Burkitt lymphoma, an aggressive form of non-Hodgkin's lymphoma. We developed a model that mimics this translocation event by inserting a mouse *Myc *cDNA gene into the immunoglobulin heavy chain locus, just upstream of the intronic Eμ enhancer. These mice, designated iMyc^Eμ^, readily develop B-cell lymphoma. To study the mechanism of Myc-induced lymphoma, we analyzed signaling pathways in lymphoblastic B-cell lymphomas (LBLs) from iMyc^Eμ ^mice, and an LBL-derived cell line, iMyc^Eμ^-1.

**Results:**

Nuclear factor-κB (NF-κB) and signal transducer and activator of transcription 3 (STAT3) were constitutively activated in iMyc^Eμ ^mice, not only in LBLs but also in the splenic B-lymphocytes of young animals months before tumors developed. Moreover, inhibition of either transcription factor in iMyc^Eμ^-1 cells suppressed growth and caused apoptosis, and the abrogation of NF-κB activity reduced DNA binding by both STAT3 and Myc, as well as Myc expression. Inhibition of STAT3 signaling eliminated the activity of both NF-κB and Myc, and resulted in a corresponding decrease in the level of Myc. Thus, in iMyc^Eμ^-1 cells NF-κB and STAT3 are co-dependent and can both regulate Myc. Consistent with this, NF-κB and phosphorylated STAT3 were physically associated with one another. In addition, LBLs and iMyc^Eμ^-1 cells also showed constitutive AKT phosphorylation. Blocking AKT activation by inhibiting PI3K reduced iMyc^Eμ^-1 cell proliferation and caused apoptosis, via downregulation of NF-κB and STAT3 activity and a reduction of Myc levels. Co-treatment with NF-κB, STAT3 or/and PI3K inhibitors led to additive inhibition of iMyc^Eμ^-1 cell proliferation, suggesting that these signaling pathways converge.

**Conclusions:**

Our findings support the notion that constitutive activation of NF-κB and STAT3 depends on upstream signaling through PI3K, and that this activation is important for cell survival and proliferation, as well as for maintaining the level of Myc. Together, these data implicate crosstalk among NF-κB, STAT3 and PI3K in the development of iMyc^Eμ ^B-cell lymphomas.

## Background

Deregulated NF-κB activity plays a critical role in the survival and radiation resistance of tumor cells in a variety of human neoplasias including B cell lymphomas (BCLs) [1-5]. NF-κB comprises a family of transcription factors that control genes implicated in B-cell activation, proliferation and resistance to apoptosis [6]. Five known, structurally conserved members of the NF-κB/Rel family function as dimers in various combinations: p50, p52, p65 (Rel A), Rel B and c-Rel. Classic NF-κB, the p50 and p65 heterodimer, is an activator of gene transcription, whereas the p50/p50 homodimer both represses and activates the transcription of target genes [7]. NF-κB exists in an inactive form in the cytoplasm because of its interaction with the inhibitory protein, IκBα [8]. NF-κB activation is controlled by the IκB kinase (IKK) complex; after stimulation by cytokines and/or growth factors, IKK phosphorylates IκB, which results in its subsequent ubiquitination and proteasomal degradation. The degradation of IκB allows NF-κB to translocate to the nucleus, where it can activate or repress target genes [9]. NF-κB not only plays a role in the survival of neoplastic B cells, but is also critical for the development and survival of normal B cells [10].

Another family of transcription factors whose members are constitutively activated in many human tumors is the STAT family. These proteins can control various cellular events such as proliferation, differentiation and cell survival [11]. One member in particular, STAT3, has been shown to be constitutively activated in a number of human tumor cell lines and primary tumors, including several hematological malignancies [12,13]. STAT3 can be activated by IL6, interferons, epidermal growth factor or leptin, through the activity of members of the receptor-associated Janus kinase (JAK) family, which comprises JAK1, JAK2, JAK3, or TYK2 [14-16]. JAKs phosphorylate STAT3 at tyrosine (Tyr)-705, leading to its dimerization and subsequent translocation to the nucleus where it activates target genes [17]. In addition, maximal transcriptional activation of STAT3 requires phosphorylation at serine (Ser)-727 in response to cytokine stimulation [18-20].

Yet another important pathway of signal transduction in B cells and B-cell neoplasms is one involving phosphatidyl inositol-3 kinase (PI3K) and AKT. Aberrant activation of this pathway is a common molecular alteration in human malignancies [21-25]. PI3K becomes activated by receptor tyrosine kinases or other cell-surface receptors, resulting in an elevation in the production of the membrane lipid phospho-inositol (3,4,5)P_3 _(PIP_3_) from phospho-inositol(4,5)P_2 _(PIP_2_). The level of PIP_3 _is negatively controlled by the phosphatase and tensin homolog (PTEN), which converts PIP_3 _back to PIP_2_. AKT binds PIP_3 _at the plasma membrane, and this leads to phosphorylation of AKT at Ser-473 in its regulatory domain. This activated form of AKT can then phosphorylate, and thereby regulate the function of, many cellular proteins that are involved in cell proliferation and survival, as well as in tumorigenesis and metastasis [26-30].

Although activation of NF-κB, STAT3 and/or the PI3K/AKT pathway in B cell neoplasms has been described [23-25], the mechanism by which these pathways contribute to the development of BCLs remains unclear, as do the circumstances under which this occurs. We recently developed the iMyc^Eμ ^mouse, an experimental model system for studying Myc-driven neoplastic transformation of B cells. Previous studies have shown that, on a mixed background of segregating C57BL/6 and 129/SvJ alleles, the iMyc transgene causes the development of various B cell-derived lymphomas: lymphoblastic B-cell lymphomas (LBLs) in 50% of the mice; diffuse large B-cell lymphomas (DLBCLs) in 25% of the mice, and plasmacytomas (PCTs) in 20% of the mice [31]. In the study described here, we investigated the role of NF-κB, STAT3 and PI3K signaling in LBL, the most prevalent tumor type in the iMyc^Eμ ^mice. We found that constitutive activation of NF-κB and STAT3 begins well before frank tumors develop, with co-activation of NF-κB and STAT3 playing a role in tumor maintenance, and activation of the PI3K/AKT pathway in the neoplastic B cells being responsible, in part, for the constitutive activation of NF-κB and STAT3. Inhibition of any one of these three pathways resulted in Myc downregulation, inhibited growth growth and promoted apoptosis in iMyc^Eμ^-LBL-derived cells. We report, for the first time, a physical association of NF-κB with STAT3 in B cells, and provide evidence for the convergence of PI3K, NF-κB and STAT3 signaling in Myc-driven lymphomagenesis.

## Materials and methods

### Tissues and cell lines

Primary LBL tumors from iMyc^Eμ ^mice [31] and the LBL-derived cell line, iMyc^Eμ^-1 [32], were used in this study. WEHI 231, RAW 8.1, and NFS-1.0 C-1 cell lines were purchased from ATCC (Rockville, MD). All cell lines were maintained in RPMI 1640 medium supplemented with 10% heat-inactivated fetal bovine serum, 200 mM L-glutamine, 50 mM 2-mercaptoethanol and antibiotics, 100 U/ml penicillin, and 100 μg/ml streptomycin (Gibco-BRL, Rockville, MD), at 37°C in a humidified 5% CO_2 _incubator. Highly enriched (>95% pure) splenic B cells were isolated from C57BL/6 (BL6) or iMyc^Eμ ^mice using CD45R (B220) microbeads and MACS^® ^separation columns (Miltenyi Biotec, Auburn, CA) according to the manufacturer's protocol. Control cultures were treated with phosphate-buffered saline (PBS) or DMSO where appropriate, and the final concentration never exceeded 0.3%.

### Preparation of nuclear and cytosolic extracts

Pellets of 10^7 ^cells or powdered-frozen LBL samples were lysed with 400 μl of 10 mM KCl, 0.2 mM EDTA, 1.5 mM MgCl_2_, 0.5 mM DTT, and 0.2 mM PMSF at 4°C for 10 minutes. The lysate was centrifuged for 5 minutes at 14,000 × *g *and supernatants were stored as cytosolic extract, at -70°C. The resulting pellet was re-suspended in 100 μl of ice-cold 20 mM HEPES (pH 7.9), 420 mM NaCl, 1.5 mM MgCl_2_, 20% (v/v) glycerol, 0.2 mM EDTA, 0.5 mM DTT, and 0.2 mM PMSF. After incubation at 4°C for 20 minutes, the lysate was centrifuged for 6 minutes at 14,000 × *g*, and the supernatant was stored as a nuclear extract, at -70°C. The concentration of cytosolic and nuclear extract was determined using a BCA kit (Bio-Rad, Richmond, CA).

### Electrophoretic mobility shift assay (EMSA) and super-shift assay

The DNA-protein binding detection kit (Gibco-BRL) was used with modifications. In brief, DNA-binding reactions were carried out in a final volume of 25 μl of buffer containing 10 mM Tris (pH 7.5), 100 mM NaCl, 1 mM DTT, 1 mM EDTA, 4% (w/v) glycerol, 0.1 mg/ml sonicated salmon sperm DNA, 10 μg of nuclear extract, and oligonucleotides. Oligonucleotides containing consensus NF-κB (Promega, Madison, WI), STAT3 (Santa Cruz Biotechnology, Santa Cruz, CA), or Myc-Max binding sites (Santa Cruz Biotechnology) were end-labeled to a specific activity of 10^5 ^CPM with γ-[^32^P]-ATP and T4-polynucleotide kinase, followed by purification on a Nick column (GE Healthcare, Piscataway, NJ). Reaction mixtures with radio-labeled oligonucleotides were incubated at room temperature for 20 minutes, and resolved on 6% non-denaturing polyacrylamide gels after addition of 2 μl bromophenol blue (0.1%). Gels were dried and subjected to autoradiography. For competition assays, 30-fold excess unlabeled oligonucleotides containing consensus or mutated NF-κB, STAT3 or Myc-Max binding sites, respectively, were added for 20 minutes at room temperature, after incubation with the radio-labeled oligonucleotides. For super-shift assays, 2 μg of antibody (Ab) was added for 20 minutes at room temperature after the initial incubation. Abs specific for p50 (sc-114X), p52 (sc-298X), p65 (sc-109X), RelB (sc-226X), c-Rel (sc-70X), Myc (sc-764X), SP-1 (sc-59X), STAT3 (sc-483X) or P-STAT3 (sc-8059X or sc-7993X) were purchased from Santa Cruz Biotechnology.

### Reverse transcription polymerase chain reaction (RT-PCR)

Semi-quantitative RT-PCR was performed by extracting total RNA using TRIzol (Sigma-Aldrich, St. Louis, MO), and this was followed by double-stranded cDNA synthesis from 1 μg of total RNA, using the AMV reverse transcriptase kit (Roche, Indianapolis, IN). Thermal cycling conditions were as follows: 95°C for 5 minutes followed by 20, 25, 30, 35, or 40 cycles (depending on the target gene) of amplification at 57°C, 72°C, and 95°C, for 1 minute each. PCR products were resolved by electrophoresis on 1% agarose gels containing ethidium bromide. Primer sequences are as follows:

PTEN, forward 5'-GGCGGTGTCATAATGTCTCTCA-3'

reverse 5'-CCCATTTTCCACTTTTTCTGAGG-3'

β-actin, forward 5'-ATGGCATTGTTACCAACTGGGACG-3'

reverse 5'-CTCTTTGATGTCACGCACGATTTC-3'.

### Whole-cell extracts and Western blotting

Whole-cell lysates were obtained by re-suspending pellets of 10^7 ^cells or powdered-frozen LBL samples in RIPA buffer (1% NP-40, 0.5% sodium deoxycholate, 0.1% SDS, 10 ng/ml PMSF, 0.03% aprotinin, 1 μM sodium orthovanadate) at 4°C for 30 minutes. Lysates were centrifuged for 6 minutes at 14 000 × *g*, and supernatants were stored at -70°C as a whole-cell extract. Total protein concentrations were determined by BCA (Bio-Rad). Western blotting was performed with 40 μg of total protein resolved by SDS-PAGE and transferred to PVDF membranes. Membranes were probed with Abs against c-Myc (sc-764), PTEN (sc-7974), or IκBα (sc-847) from Santa Cruz Biotechnology, ERK1/2 (9102), P-ERK1/2 (4377), p38 (9212), P-p38 (9211), AKT (9272), P-AKT (9271), P-AKT (9275), p70S6K (9202), or P-p70S6K (9205) from Cell Signaling (Danvers, MA), α-tubulin (T6074) or β-actin (A5316) from Sigma-Aldrich. Proteins were visualized using horseradish peroxidase-conjugated secondary Ab (1:5000) and the ECL detection kit from Amersham (GE Healthcare). To confirm equal loading, membranes were stripped and re-probed using an Ab specific for α-tubulin or β-actin. Total cell extracts from UV-treated HeLa and NIH 3T3 cells were used as positive controls for P-ERK1/2 (sc-2221) and P-p38 (sc-2210), respectively (Santa Cruz Biotechnology). Total cell extract from insulin-treated MCF-7 cells was used as a positive control for P-p70S6K (9203) (Cell Signaling).

### Proliferation assay

Proliferation was determined using the Cell Titer 96^® ^MTS/PMS assay (Promega). Briefly, 3 × 10^4 ^cells were re-suspended in 100 μl growth medium and plated into 96-well plates (Costar, Cambridge, MA). After 20 hours at 37°C and 5% CO_2_, 20 μl of MTS/PMS solution was added to each well and cells were incubated for another 4 hours before the absorbance at 490 nm was measured on a Multiskan Spectrum (Thermo Scientific, Hudson, NH).

### Apoptosis assay

Apoptosis was evaluated using the DNA fragmentation assay and fluorescence activated cell sorting (FACS)-based analysis of propidium iodide (PI), annexin V, and Caspase-3 reactivity. For DNA fragmentation, DNA was extracted using the Puregene kit (Gentra Systems, Minneapolis, MN) and resolved by electrophoresis on 1.0% agarose gels containing ethidium bromide. For the identification of cells with sub-G0/G1 DNA content, cells were resuspended in PI/Rnase buffer (BD Pharmingen, San Diego, CA) for 20 minutes at 37°C in the dark before FACS analysis. Annexin-V reactivity was determined by applying a phycoerythrin (PE)-labeled Ab (BD Pharmingen) to cells co-stained with 7-amino-actinomycin D (7-AAD). Activated caspase-3 was detected using a FITC- or PE-conjugated Ab (BD Pharmingen).

### Pharmacological inhibitors

The following small-molecule inhibitors were used: LY294002 (LY) (Promega); Lactacystin (LC), PD98059 (PD), SB203580 (SB), and rapamycin (Rap) (Biomol, Plymouth Meeting, PA); WHI P-131 (WHI) and AG 490 (Biosource, Carlsbad, CA); and AEG 3482 (AEG) (Tocris, Ellisville, MO).

### Co-Immunoprecipitation (Co-IP)

Co-IP was performed using the Universal Magnetic Co-IP kit according to the manufacturer's protocol (Active Motif, Carlsbad, CA). Briefly, 500 μg of nuclear extract was incubated with 5 μg of phosphorylated STAT3 (P-STAT3) Ab (sc-7993), NF-κB p50 Ab (sc-114), or Rabbit IgG control (sc-2345) for 3 hour at 4°C. 25 μl of Protein G Magnetic Beads were added to each tube and then incubated for 1 hour at 4°C. Immunoprecipitates were washed 4 times each with 500 μl wash buffer using a magnetic stand, after which the pellets were resuspended with 2 × reducing loading buffer. Western blot analysis was performed using a primary NF-κB p50 Ab (sc-114) or P-STAT3 Ab (sc-7993), respectively.

### IL6 and IL10 assays

IL6 or IL10 expression was assesssed using the RayBio Mouse Cytokine Antibody Array III kit (RayBiotech, Norcross, GA) or the Mouse IL-6 or IL-10 Enzyme-Linked Immunosorbent Assay (ELISA) kit (eBioscience, San Diego, CA), according to the manufacturer's protocol. Samples tested were 10^7 ^splenic B or B220-negative cells from 2-month-old BL6 or iMyc^Eμ ^mice, separated by CD45R (B220) microbeads and MACS^® ^separation columns (Miltenyi Biotec).

## Results

### NF-κB and STAT3 are constitutively activated in B-cell lymphomas of iMyc^Eμ ^mice

**Figure 1:**
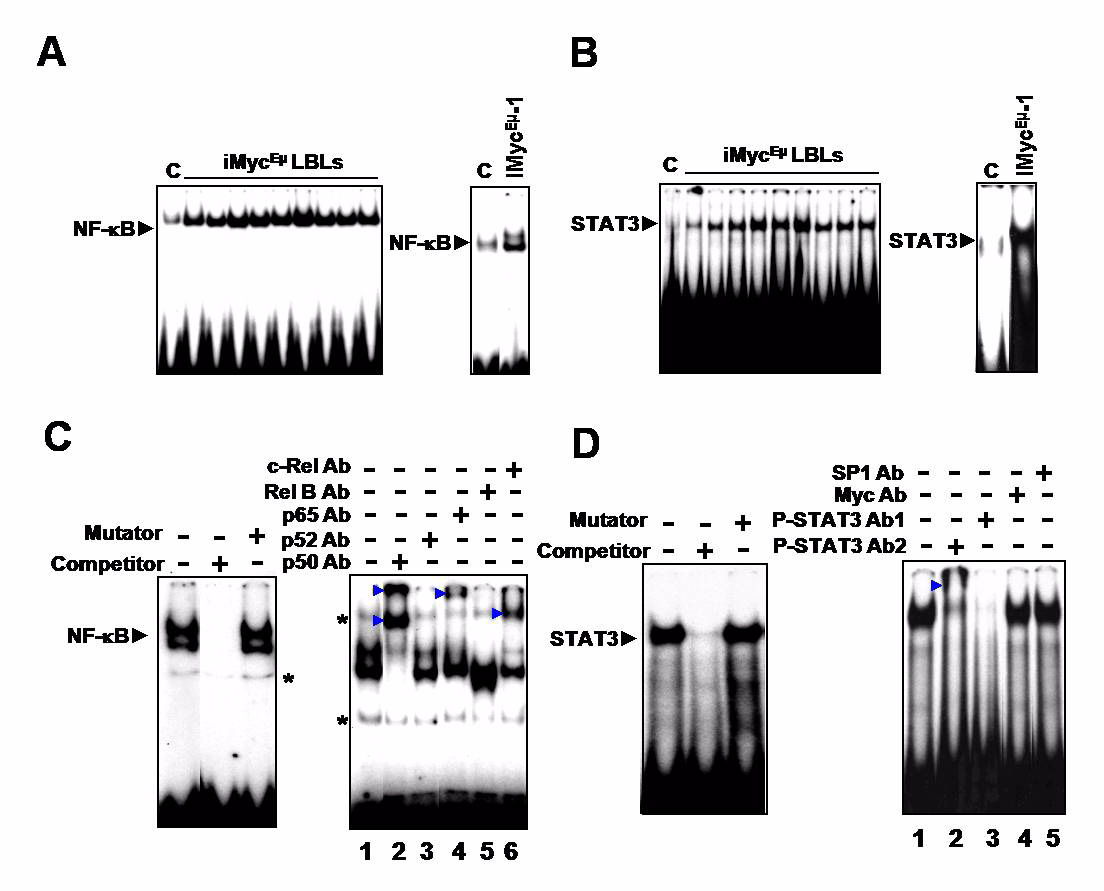
**NF-κB and STAT3 are constitutively activated in LBLs and iMyc^Eμ^-1 cells**. (A and B) EMSA using an NF-κB-specific probe (A) and a STAT3-specific probe (B), respectively, showing constitutive DNA-binding by NF-κB and STAT3 in LBL tumors (left panel), iMyc^Eμ^-1 cells (right panel) and control (C) C57BL/6 splenic B cells. (C) EMSA competition assay (left panel) demonstrating specificity of NF-κB probe. Right panel shows a super-shift assay for NF-κB, using antibodies (Abs) specific for the denoted subunits. Asterisks denote non-specific bands that are covered by the super-shifted bands in lanes 2 and 6. (D) Competition (left panel) and super-shift (right panel) assays for STAT3. Competitor is an unlabelled oligonucleotide probe, and mutator is an unlabelled probe with a mutation that abrogates DNA-binding. P-STAT3 super-shift Abs are both specific for phosphorylated STAT3 at Tyr-705; Ab 1 is sc-7993X and Ab 2 is sc-8059X. SP1 and Myc Abs were used as negative controls. Arrowheads denote shifted bands. Images are representative, and image splicing was carried out only for the same experiment, the same gel and the same exposure times.

Both NF-κB and STAT3 are important for the proliferation and survival of normal B cells and several types of non-Hodgkin's lymphoma (NHL) [33-37]. We used EMSA to examine NF-κB and STAT3 activity in both iMyc^Eμ^-derived LBLs and the iMyc^Eμ^-1 cell line. All nine LBLs and the iMyc^Eμ^-1 cells showed abnormal activation of both NF-κB (Figure 1A) and STAT3 (Figure 1B) when compared to isolated splenic B cells from control C57BL/6 (BL6) mice.

To ascertain the specificity and subunit composition of NF-κB, we conducted competition and super-shift assays on iMyc^Eμ^-1 cells. Incubation of nuclear extracts with 30-fold excess unlabelled competitor probe abolished the constitutive NF-κB activity, but incubation with unlabelled probes containing a mutation that disables NF-κB binding (mutator) did not (Figure 1C, left), indicating that the observed band was indeed NF-κB. Super-shift assays were carried out using antibodies (Ab) against NF-κB subunit p 50, p 52, p 65, Rel B, or c-Rel. As shown in the right panel of figure 1C, notable shifts were observed when antibodies against p50 (lane 2), p 65 (lane 4) or c-Rel (lane 6) were added. The p50 Ab shifted both NF-κB-specific bands to higher molecular-weight positions, whereas the p 65 and c-Rel antibodies shifted only the upper band. Neither the p 52 nore the RelB antibody produced any shift. These results indicate that the constitutively activated NF-κB in iMyc^Eμ^-1 cells is likely comprised of p 50/p 50 homodimers and/or p 50/p 65 and p 50/c-Rel heterodimers. That the observed shift involving p65 was less pronounced suggests that p 50/p 50 and p 50/c-Rel complexes predominate.

Competition and super-shift assays were also performed for STAT3. Incubation of nuclear extracts with competitor abrogated the constitutive STAT3 activity, whereas the addition of mutator did not (Figure 1D, left). Incubation with one Ab specific for STAT3 phosphorylated at Tyr-705 shifted the band to a higher molecular weight, and incubation with another Ab completely eliminated the STAT3 band (Figure 1D, right, lanes 2 and 3, respectively). These results show that the activated form of STAT3 is phosphorylated on Tyr-705. Myc Ab and SP1 Ab were used as negative controls and did not show any change.

### Constitutive activation of NF-κB and STAT3 occurs early in iMyc^Eμ ^mice

**Figure 2:**
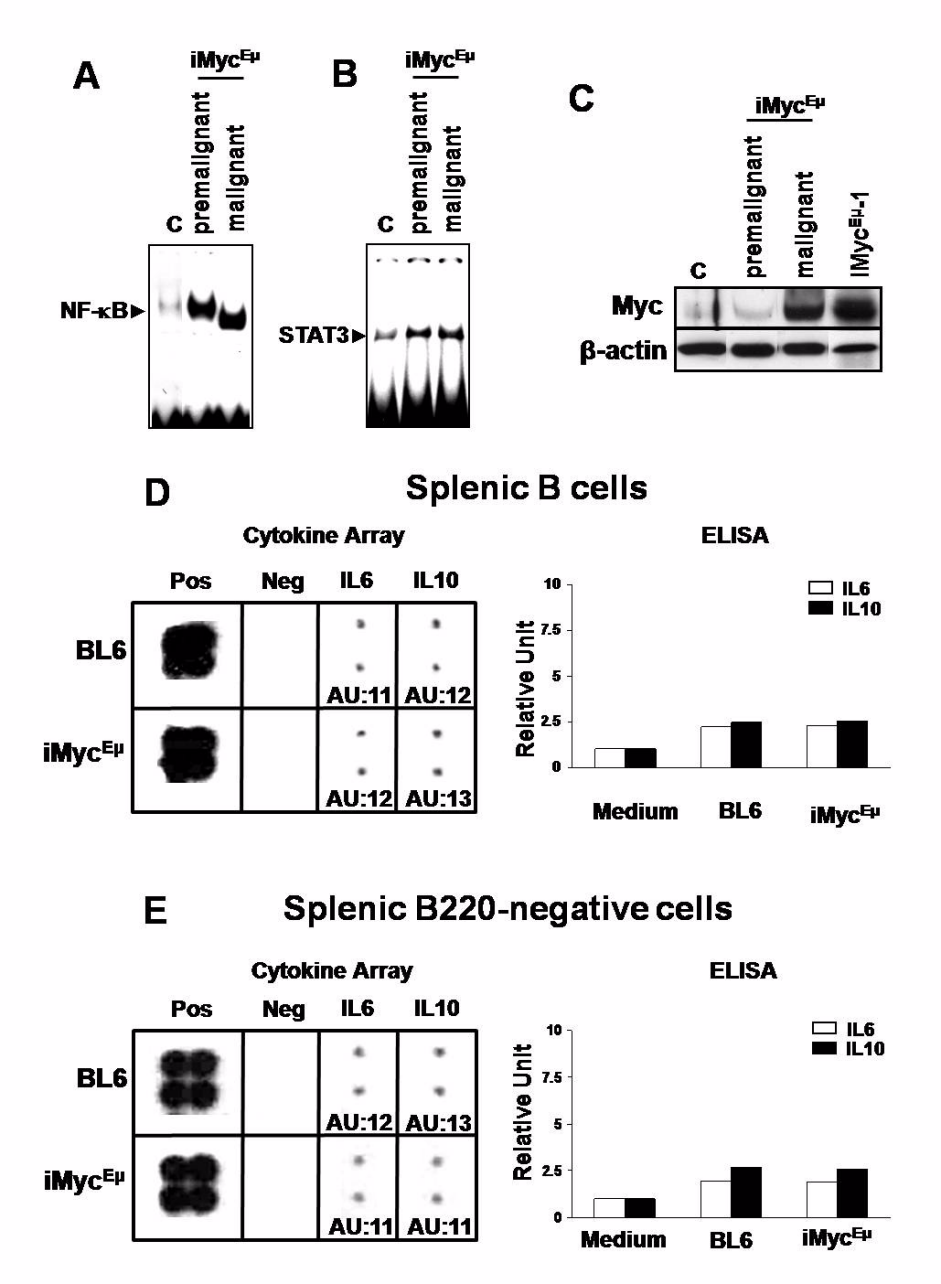
**Constitutive activation of NF-κB and STAT3 in iMyc^Eμ ^mice occurs early**. (A and B) EMSA showing elevated DNA-binding activity of NF-κB (A) and STAT3 (B), respectively, in both premalignant (average spleen weight 80-150 mg) and malignant (average spleen weight ~750 mg) splenic B220-positive B cells from iMyc^Eμ ^and control BL6 (average spleen weight 80-100 mg) mice. (C) Western blot showing the level of Myc protein in control, premalignant and malignant splenic B cells, defined as above, and in iMyc^Eμ^-1 cells. β-actin was used as a loading control. (D and E) Cytokine array (Left panel) and ELISA (Right panel) for IL6 and IL10, showing the levels of IL6 and IL10 protein in splenic B cells (D) and in splenic B220-negative cells (E) from 2-month old control or iMyc^Eμ ^mice. ImageQuant software was used to quantitate the results of cytokine array, and data are expressed as arbitrary densitometric units (AU) after normalization to positive controls (Pos). "Neg" denotes negative controls.

The use of mouse models offers a valuable opportunity to study early events that contribute to tumor development. To determine whether NF-κB and STAT3 activation occurred before tumors were present, we examined NF-κB and STAT3 activity in splenic B cells from tumor-free (premalignant) or tumor-bearing (malignant) iMyc^Eμ ^mice, using splenomegaly and age as two independent indicators of tumor progression. As expected, NF-κB (Figure 2A) and STAT3 (Figure 2B) activity was increased in splenic B cells isolated from mice with malignant growths (i.e. mice with spleen masses of on average 750 mg) relative to that in splenic B cells from normal BL6 mice (i.e. spleen masses between 80-100 mg). However, splenic B cells from iMyc^Eμ ^mice with no visible signs of malignancy and spleen masses between 80-150 mg, which were considered premalignant, also had abnormally high NF-κB and STAT3 activity. Similarly, splenic B cells from one to four month-old premalignant iMyc^Eμ ^mice (as defined without regard to spleen weight) exhibited highly elevated NF-κB and STAT3 DNA-binding activity, at as early as one month of age, relative to splenic B cells from age-matched, normal BL6 mice (see additional file 1A and 1B). These data show that constitutive activation of both NF-κB and STAT3 occurs months before tumors are present, and at an early age, in iMyc^Eμ ^mice [31].

We also evaluated the level of Myc protein in splenic B cells of premalignant and malignant iMyc^Eμ ^mice (as defined by spleen size), as well as in iMyc^Eμ^-1 cells (Figure 2C). It is widely accepted that the cellular level of Myc must remain exquisitely titrated to induce neoplastic development but avoid apoptosis [38]. Consistent with this, only a marginal elevation of Myc protein was repeatedly observed in premalignant iMyc^Eμ ^B splenocytes. Myc protein was, however, dramatically elevated in malignant B cells and in iMyc^Eμ^-1 cells. Even though NF-κB and STAT3 are known to drive Myc expression [34,38-41], constitutive activity of NF-κB and STAT3 is not sufficient to increase the level of Myc at premalignancy in iMyc^Eμ ^B cells.

IL6 and IL10 are important cytokines that have been implicated in lymphomagenesis and are linked to NF-κB and STAT3 signaling through autocrine and/or paracrine loops [4,14,39]. We performed cytokine array and ELISA to examine whether elevated expression of IL6 and/or IL10 are involved in early activation of NF-κB and STAT3 in iMyc^Eμ ^mice. As shown in Figure 2D, no significant difference was observed in the level of either IL6 or IL10 between the splenic B cells of BL6 and premalignant iMyc^Eμ ^mice, suggesting that elevated levels of IL6 and IL10 are not responsible for elevated NF-κB or STAT3 activity through autocrine signaling. IL6 and IL10 expression was also nearly equivalent in splenic B220-negative cells from premalignant iMyc^Eμ ^and control mice (Figure 2F), suggesting that IL6 and IL10 are not upregulated in the B-cell microenvironment. Additionally, we independently evaluated the levels of IL6 and IL10 in LBL tumors using RT-PCR, GEArray (SA Bioscience, Frederick, MD) and Affymetrix GeneChip Arrays (Affymatrix, Santa Clara, CA) (n = 11, 8 and 4, respectively). No elevation of IL6 and IL10 expression has been observed in these iMyc^Eμ ^tumors compared to normal BL6 splenic B cells (data not shown). These data suggest that the overexpression of IL6 and IL10 does not occur as a response to elevated NF-κB or STAT3 activity, nor as a cause thereof, through either autocrine or paracrine signaling in iMyc^Eμ ^mice.

### Inhibition of NF-κB in iMyc^Eμ^-1 cells reduces cell proliferation, causes apoptosis, and downregulates STAT3 activity and Myc expression

**Figure 3:**
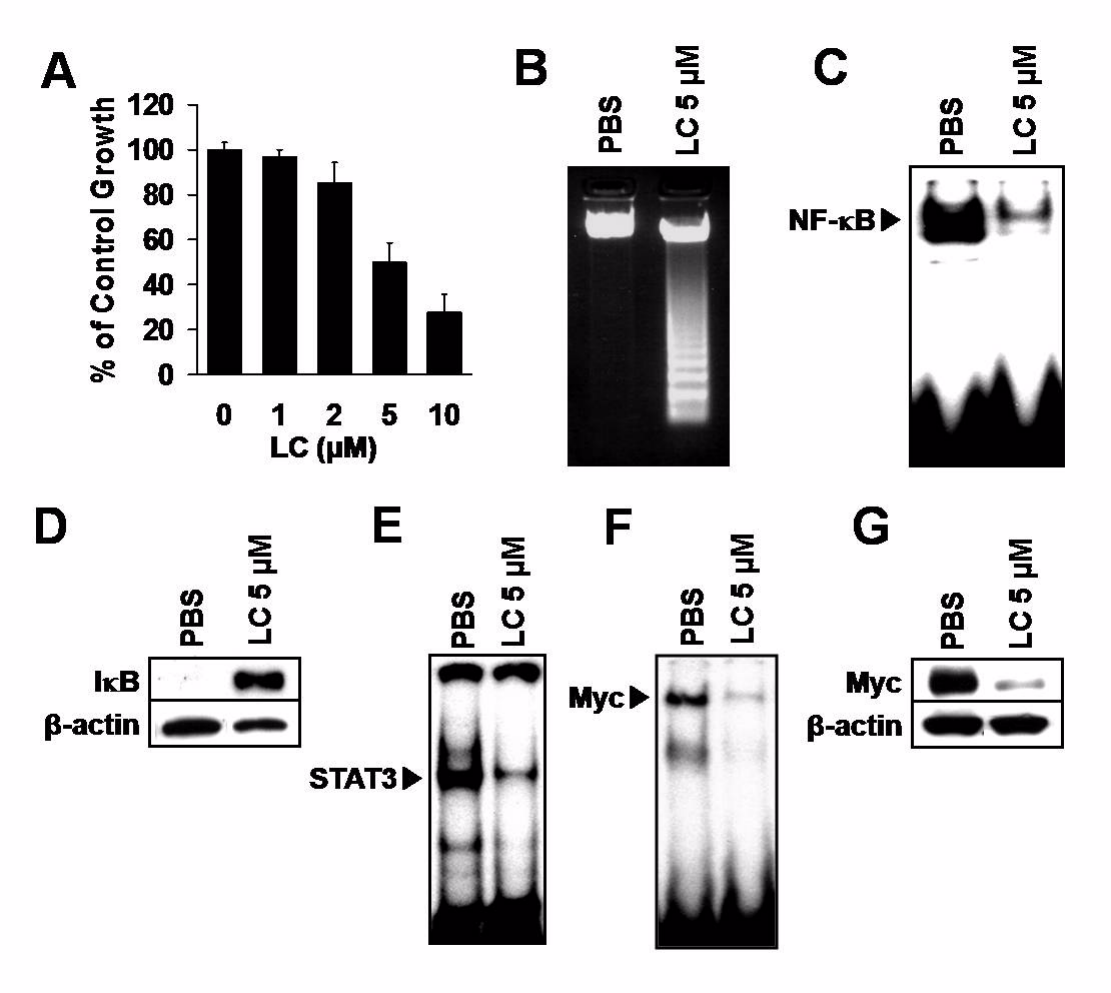
**NF-κB inhibition suppresses growth, causes apoptosis and downregulates STAT3 and Myc activity in iMyc^Eμ^-1 cells**. (A) MTS/PMS cell proliferation assay, after culture with the NF-kB inhibitor lactacystin (LC) at various concentrations as indicated. Data were normalized to vehicle control, and error bars represent the standard deviation from a representative experiment performed in triplicate. (B) Agarose gel showing DNA fragmentation in sample treated with LC, but not in PBS control. (C) EMSA showing reduced NF-κB DNA-binding after LC treatment. (D) Stabilization of IκB protein after treatment with LC, as determined by Western blotting. (E) Reduced STAT3 DNA-binding activity after NF-κB inhibition, as observed by EMSA. (F) EMSA showing that Myc DNA-binding activity is reduced after LC treatment. (G) Western blot showing that Myc protein levels are reduced after NF-κB inhibition. β-actin was used as a loading control for Western blots. All LC incubations were for 24 hours.

To investigate the role of NF-κB in proliferation and survival, we cultured iMyc^Eμ^-1 cells in the presence of the NF-κB inhibitor, Lactacystin (LC). LC treatment for 24 hours inhibited growth of iMyc^Eμ^-1 cells in dose-dependent fashion, as measured by MTS (Figure 3A). DNA laddering indicated that LC also induced apoptosis (Figure 3D). By EMSA, we confirmed that 5 μM LC inhibited NF-κB activity (Figure 3C) by stabilizing IκB (Figure 3D). Notably, other NF-κB inhibitors, BAY-11 7085 or Helenin, which function by blocking IκB phosphorylation or preventing DNA-binding by NF-κB, respectively, had similar inhibitory effects on the proliferation of iMyc^Eμ^-1 cells (data not shown). We then examined whether inhibiting NF-κB altered STAT3 or Myc activity. As shown in Figure 3E and 3F, treatment with LC dramatically reduced the activity of both STAT3 and Myc. The reduction in Myc activity corresponded to a remarkable decrease in the level of Myc protein (Figure 3G). EMSA competition and super-shift assays were done as before, to demonstrate the specificity of Myc DNA-binding (see additional file 2). These data imply that NF-κB is necessary for the proliferation and survival of iMyc^Eμ^-1 cells, and to link NF-κB to the activities of STAT3 and Myc.

### STAT3 is required for optimal proliferation and survival of iMyc^Eμ^-1 cells, and is linked to activation of NF-κB and Myc

**Figure 4:**
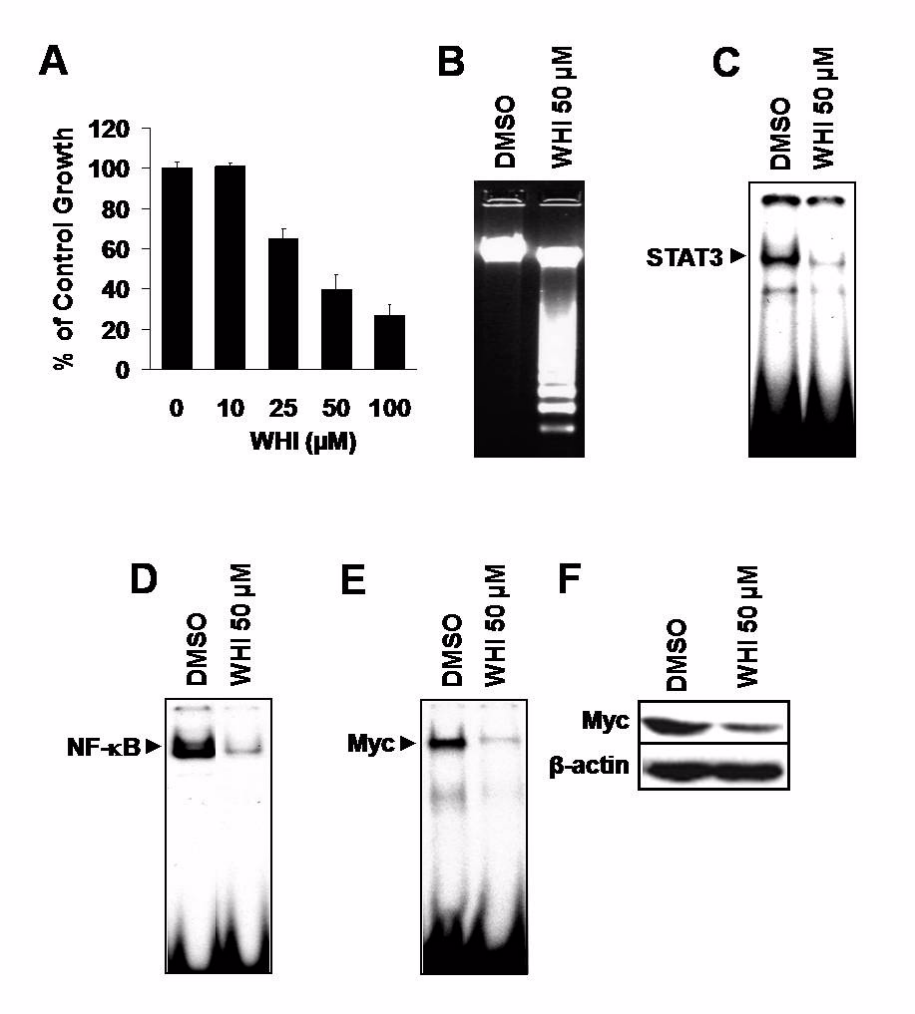
**STAT3 inhibition reduces growth, leads to apoptosis and downregulates NF-κB and Myc activity in iMyc^Eμ^-1 cells**. (A) Dose-dependent suppression of proliferation (MTS/PMS) after culture with the STAT3 inhibitor WHI P-131 (WHI) at various concentrations, as indicated. Data were normalized to DMSO treatment controls, and error bars represent the standard deviation for a representative experiment performed in triplicate. (B) Agarose gel showing DNA fragmentation after treatment with WHI. (C, D and E) EMSA revealing reduced STAT3 (C), NF-κB (D) and Myc (E) DNA-binding, respectively, after WHI treatment. (F) Western blot showing a decrease in the level of Myc protein after STAT3 inhibition. β-actin was used as a loading control. All WHI incubations were for 24 hours.

STAT3 was also constitutively activated in iMyc^Eμ ^LBLs, so we examined whether signaling through this transcription factor is important for the proliferation and survival of iMyc^Eμ^-1 cells. Cells were cultured in the presence of the potent JAK3/STAT3 specific inhibitor WHI P-131 (WHI), and this suppressed growth in a dose-dependent manner (Figure 4A) and ultimately led to apoptosis (Figure 4B) through abrogation of STAT3 activity (Figure 4C). Use of the potent JAK2/STAT3-specific inhibitor AG 490 resulted in similar inhibitory effects on the proliferation of iMyc^Eμ^-1 cells (data not shown). We then assessed whether STAT3 signaling had an effect on NF-κB and/or Myc activity. Inhibiting STAT3 severely reduced the DNA binding activity of both NF-κB (Figure 4D) and Myc (Figure 4E), and led to a reduction in Myc protein levels (Figure 4F). Like NF-κB, STAT3 appears to be necessary for the proliferation and survival of iMyc^Eμ^-1 cells. Thus STAT3 is reciprocally linked to NF-κB activity and has similar effects on Myc, a finding that intimates a co-dependency between NF-κB and STAT3 signaling.

### NF-κB and phosphorylated STAT3 associate physically in iMyc^Eμ^-1 cells

**Figure 5:**
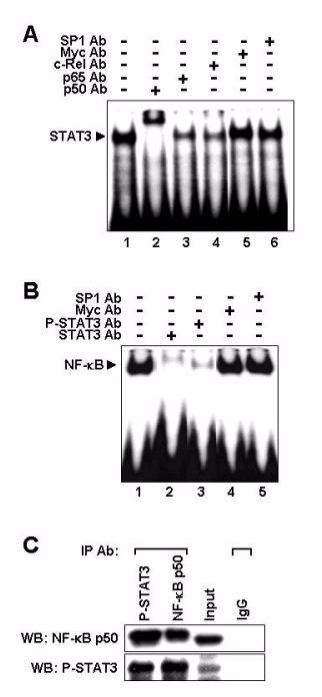
**NF-κB and STAT3 associate with one another physically in iMyc^Eμ^-1 cells**. (A and B) EMSA super-shift assays performed with STAT3-specific probes and NF-κB-specific Abs (A), or NF-κB-specific probes and STAT3-specific Abs (B). Abs were specific for NF-κB subunits, Tyr-705 phosphorylated STAT3 (P-STAT3) and total STAT3, as indicated. Abs against SP1 and Myc were used as negative controls. (C) Co-IP and Western blot showing co-immunoprecipitation of NF-κB p50 and P-STAT3. Abs used for immunoprecipitations (IP) and Western blotting (WB) are designated. Images are representative, and image splicing was only carried out only for the same experiment, the same gel and the same exposure times.

Recent studies have shown that NF-κB and STAT3 physically associate with one another in several cell types [42-46]. Our findings indicate that constitutively activated NF-κB and STAT3 may cooperatively regulate each other. Thus, we investigated whether STAT3 and NF-κB are physically associated in iMyc^Eμ^-1 cells. Super-shift assays were performed with a STAT3-specific oligonucleotide probe and antibodies specific for p 50, p 65, or c-Rel NF-κB subunits. As shown in Figure 5A, our results showed a clear shift in DNA-bound STAT3 when a p 50 Ab was added (lane 2). Addition of a p 65 Ab (lane 3) or c-Rel Ab (lane 4) led to a slight decrease in band intensity (~1/3 of control). This suggests that p65 and c-Rel may be involved in the complex, consistent with our previous observation of shifts in NF-κB DNA-binding with these subunits (see Figure 1C). In the reciprocal experiment, only the addition of an anti-STAT3 Ab (Figure 5B, lane 2) or a P-STAT3 (Tyr 705) Ab (lane 3) affected DNA-binding of NF-κB. These super-shift results indicate that NF-κB and P-STAT3 are physically associated. For further verification, we performed Co-IP and Western blotting for P-STAT3 or the p50 subunit of NF-κB. In keeping with the super-shift results, NF-κB and P-STAT3 were co-immunoprecipitated (Figure 5C). Thus, NF-κB and STAT3 reside in the same complex in iMyc^Eμ^-1 cells.

### AKT is aberrantly activated in LBLs and iMyc^Eμ^-1 cells

**Figure 6:**
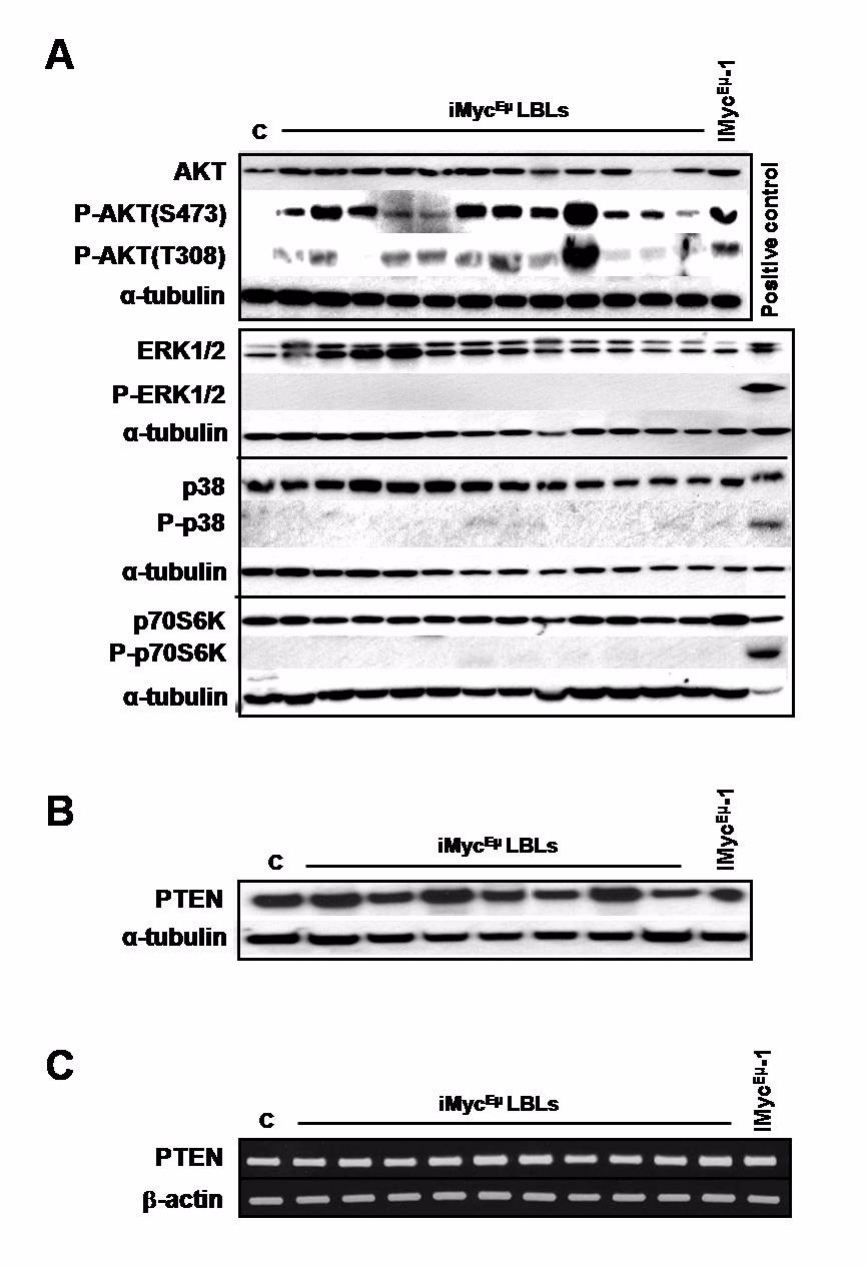
**AKT is constitutively phosphorylated, in a PTEN independent-manner, in a majority of LBLs and iMyc^Eμ^-1 cells**. (A) Western blot analysis of the activating-phosphorylation status of key proteins of the PI3K (AKT, P-AKT S473 and T308), MAPK (ERK 1/2, P-ERK1/2, total p 38, P-p 38) and mTOR (p70S6K, P-p70S6K) signaling pathways. Positive controls for P-ERK1/2, P-p38 and P-p70S6K were from extracts of UV-treated HeLa cells, NIH 3T3 cells and insulin-treated MCF-7 cells, respectively. (B and C) Levels of PTEN protein (B) and mRNA (C) in LBLs and iMyc^Eμ^-1. α-tubulin and β-actin served as loading controls, respectively. "C" denotes control BL6 splenic B cells.

Having observed signaling crosstalk between constitutively activated NF-κB and STAT3, we investigated the role of some other major signaling pathways in LBLs and iMyc^Eμ^-1 cells. Given that PI3K, mTOR and MAPK signaling are important for cell survival and proliferation [21-25], we examined activation of these pathways. The PI3K downstream effector AKT was phosphorylated on both Ser-473 and threonine (Thr)-308 in nearly all LBLs and iMyc^Eμ^-1 cells, indicating that it was constitutively activated (Figure 6A). In contrast, phosphorylated forms of ERK, p38 and p70S6K were not readily apparent, indicating that the MAPK and mTOR signaling pathways were not activated. In many types of tumors, the loss or mutation of PTEN leads to elevated activity of the PI3K/AKT pathway [47]. Thus, we evaluated the PTEN levels in LBLs and iMyc^Eμ^-1 cells by Western blotting and RT-PCR. PTEN protein or mRNA remained unchanged compared to levels in normal splenic B cells (Figure 6B and 6C, respectively). Activation of AKT from these particular tumor samples and quantitation of PTEN mRNA are shown in additional file 3. Sequencing of PTEN showed no mutation in the *Pten *gene in either LBLs or iMyc^Eμ^-1 cells (data not shown). Additionally, because activating mutations of PIK3CA can result in the constitutive phosphorylation and activation of AKT [48], we sequenced the *Pik3ca *gene. However, we did not find mutations in this gene in any LBLs or iMyc^Eμ^-1 cells (data not shown). These results suggest that constitutive activation of the AKT, but not mTOR or MAPK, pathways is involved in the pathogenesis of iMyc^Eμ ^lymphoma, independent of loss or mutation of either *Pten *or *Pik3ca*.

### PI3K/AKT is important for the proliferation and survival of iMyc^Eμ^-1 cells and is linked to the NF-κB and STAT3 activation, as well as to Myc regulation

**Figure 7:**
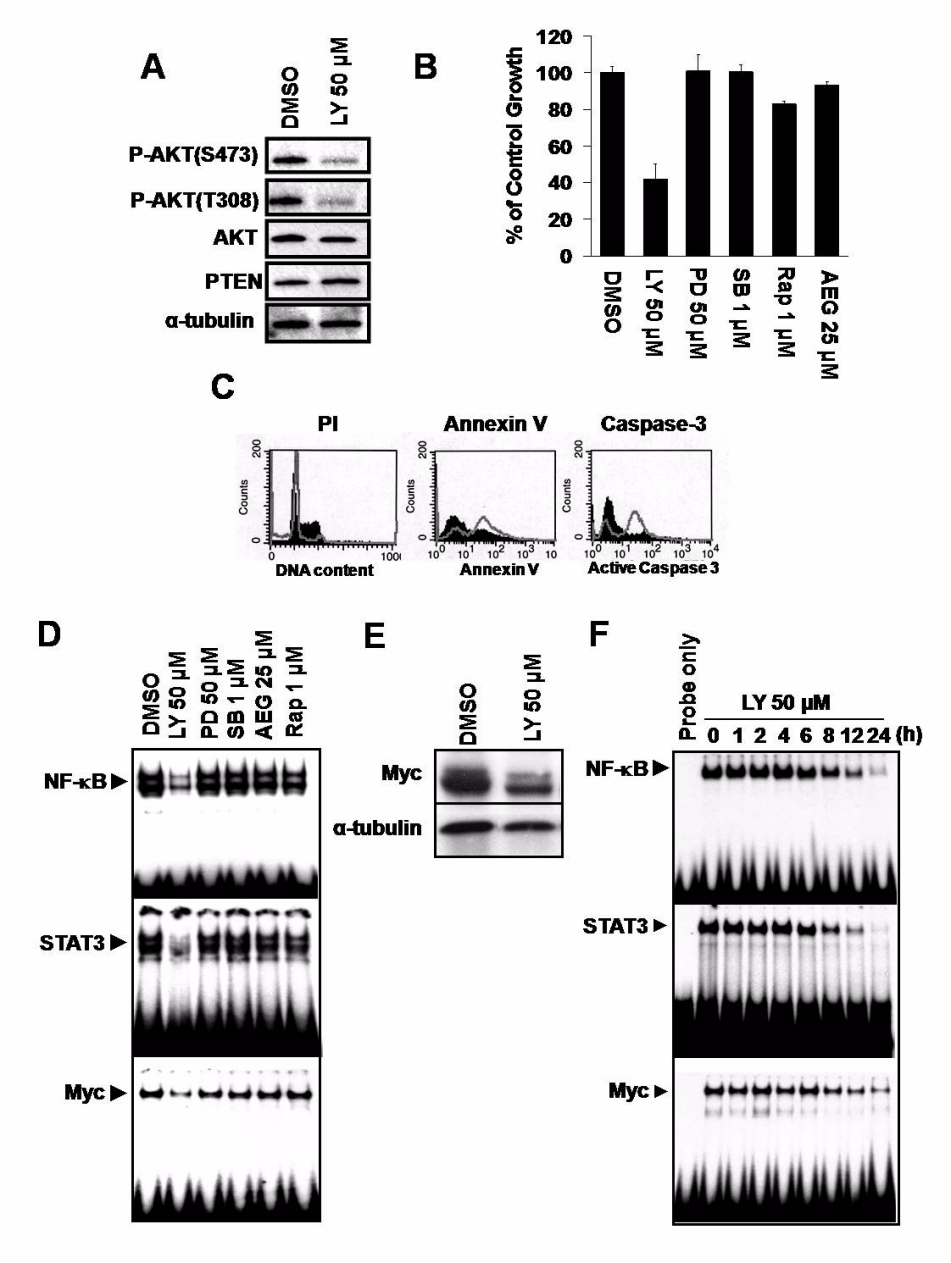
**PI3K inhibition diminishes NF-κB, STAT3 and Myc activity in iMyc^Eμ^-1 cells, and reduces their proliferation and survival**. (A) Western blot showing the levels of total AKT, PTEN and phosphorylated AKT (S473 and T308) after treatment with LY294002 (LY). α-tubulin was used as a loading control. (B) MTS/PMS assay after treatment with vehicle control, LY, PD98059 (PD), SB203580 (SB), rapamycin (Rap), or AEG 3482 (AEG) at the indicated concentrations. Data were normalized to DMSO-treatment controls, and error bars represent the standard deviation from a representative experiment performed in triplicate. (C) Representative FACS analyses on LY- (open grey histogram) or DMSO- (filled black histogram) treated cells showing an increase in sub-G0/G1 DNA, as assessed by propidium idodide (PI) staining (left panel), and apoptosis as assessed by increases in both Annexin V (middle panel) and activated caspase 3 (right panel) staining. (D) EMSA showing reduced DNA-binding activity of NF-κB, STAT3 and Myc after treatment with LY, but not PD, SB, AEG or Rap. (E) Western blot demonstrating reduced Myc protein levles after inhibition of PI3K; α-tubulin served as a loading control. (F) NF-κB, STAT3 and Myc DNA-binding activity is reduced in a time-dependent manner after PI3K is inhibited with LY. The incubation time with small-molecule inhibitors was 24 hours unless otherwise indicated.

To determine whether constitutive activation of the PI3K/AKT pathway plays a critical role in the proliferation and survival of iMyc^Eμ^-1 cells, we cultured them in the presence of the PI3K inhibitor LY294002 (LY). Treatment with LY substantially reduced phosphorylation of AKT (Figure 7A), and resulted in growth suppression (Figure 7B, see additional file 4A) and apoptosis (Figure 7C, see additional file 4B). In keeping with the Western blot results (see Figure 6A), inhibition of ERK by PD98059 (PD), of p38 by SB203580 (SB), of mTOR by rapamycin (Rap), or of JNK by AEG 3482 (AEG) had a marginal to no effect on iMyc^Eμ^-1 cell proliferation (Figure 7B, see additional file 4B and 4C). These results show that the PI3K/AKT pathway, but not the MAPK or mTOR pathways, plays an important role in the proliferation and survival of iMyc^Eμ^-1 cells. The requirement of PI3K/AKT signaling for constitutive activation of NF-κB, STAT3 and Myc was then examined by EMSA. Inhibition of PI3K significantly reduced NF-κB, STAT3 and Myc activity (Figure 7D) and also led to a reduction of Myc protein (Figure 7E). These effects were identical to those seen following the inhibition of either NF-κB (see Figure 3) or STAT3 (see Figure 4) alone, strongly suggesting crosstalk amongst PI3K/AKT, NF-κB and STAT3.

So far our studies had only looked at a snapshot of transcription-factor activity, so we evaluated whether the activity of NF-κB, STAT3 and/or Myc were temporally regulated as a result of PI3K signaling in iMyc^Eμ^-1 cells. Differential timing could hint at the order in which these transcription factors might influence one another. The DNA-binding activity of NF-κB and STAT3 diminished with identical kinetics, beginning about six hours after treatment with LY (Figure 7F). Notably, the inhibition of Myc activity was delayed by about two hours compared to inhibition by NF-κB and STAT3 (Figure 7F). These results are in harmony with the possibility that signaling progresses from PI3K to NF-κB and STAT3, which then regulate Myc.

### PI3K, NF-κB and/or STAT3 inhibitors have an additive, rather than synergistic, inhibitory effect on iMyc^Eμ^-1 cell proliferation

**Figure 8:**
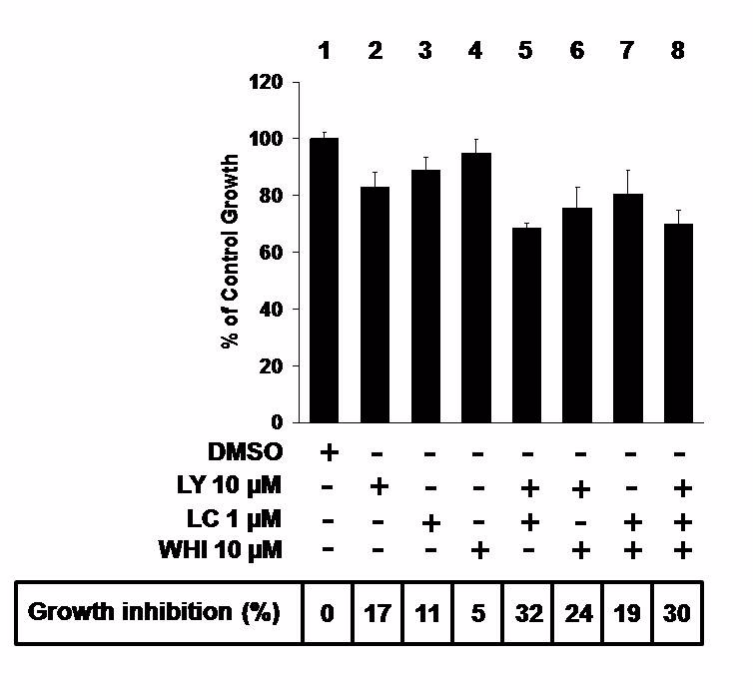
**Co-treatment with small-molecule inhibitors of NF-κB, STAT3 and/or PI3K additively inhibits the proliferation of iMyc^Eμ^-1 cells**. MTS/PMS cell proliferation assay after 24-hour treatment with low doses of LY, LC and WHI in isolation or in various combinations, as indicated. Box at the bottom gives the average percent (%) growth inhibition. Data were normalized to DMSO-treatment controls, and error bars represent the standard deviation from a representative experiment performed in triplicate.

Co-treatment with inhibitors of different signaling pathways can provide useful information regarding intracellular pathway linkage and signal transduction. Because our results have shown that inhibition of any one pathway - PI3K, NF-κB or STAT3 - suppresses proliferation and causes apoptosis, we tested whether co-treatment with inhibitors against these pathways leads to synergitic effects, as has been reported for to be the case for NF-κB and STAT3 [49]. Synergism between these inhibitors would indicate that the target genes (not evaluated here) elicited by NF-κB and STAT3 individually have a greater effect on cell survival and proliferation than the set of target genes elicited by convergent NF-κB/STAT3 signaling. To test this possibility, we cultured iMyc^Eμ^-1 cells with low doses of LC, WHI or LY, which individually cause only a very weak or a modest inhibition of proliferation (Figure 8, lanes 2-4). Regardless of the co-treatment combination, an additive, rather than synergistic, effect was observed (Figure 8, compare lane 5 to lanes 2 and 3, lane 6 to lanes 2 and 4, lane 7 to lanes 3 and 4). Considering that there is a certain dependence of both NF-κB and STAT3 on PI3K signaling (see Figure 7), and that NF-κB and STAT3 are physically located in the same molecular complex (see Figure 5), these results suggest that PI3K, NF-κB and STAT3 converge in Myc-driven lymphoma.

## Discussion

An enhanced understanding of the signal transduction pathways underlying the development of B-cell neoplasms is an important step towards identifying novel targets for tumor therapy and prevention. Although previous studies have demonstrated that NF-κB, STAT3 and/or PI3K play critical roles in growth control, survival, and chemotherapy resistance of B-cell and plasma-cell neoplasms [50-52], the precise function of NF-κB, STAT3 and/or PI3K in the development of these tumors is not completely understood. In this study, we used the iMyc^Eμ ^LBL model to uncover signaling crosstalk between NF-κB, STAT3 and PI3K signaling. To our knowledge, this is the first report of crosstalk amongst these pathways in B lymphoma cells. We found that constitutive activation of the PI3K/AKT, but not the mTOR or MAPK pathways, was found to be at least partially responsible for aberrant NF-κB and STAT3 activity. Inhibition of NF-κB, STAT3 or PI3K signaling in iMyc^Eμ ^B cells, respectively, led to growth suppression, apoptosis and downregulation of Myc. Combined inhibition had an additive effect on proliferation, suggesting that NF-κB and STAT3 converge downstream of PI3K. Our finding that NF-κB and STAT3 are physically associated in iMyc^Eμ^-1 B cells supports this interpretation. Signaling crosstalk of NF-κB, STAT3 and PI3K may play an important role in Myc-induced B-cell lymphoma in mice.

The finding that NF-κB, STAT3 and PI3K are constitutively activated in LBLs and iMyc^Eμ^-1 cells is in keeping with the aberrant activity of these pathways observed in various types of B cell neoplasms. Constitutive activation of NF-κB has frequently been observed in follicular lymphoma [53,54], DLBCL [55], mucosa-associated lymphoid tissue (MALT) lymphoma [56], multiple myeloma (MM) [57], and mantle-cell lymphoma (MCL), as well as MCL cell lines, in which inhibition of this constitutive activation induces growth arrest and apoptosis [58-60]. Aberrant STAT3 activation has been documented in MM [61], Hodgkin's disease [62], anaplastic lymphoma kinase-positive (ALK) DLBCL [63], and activated B-cell (ABC) DLBCL, in which JAK2/STAT3 inhibitors trigger arrest and apoptosis [40]. Activation of the PI3K pathway is one of the most common defects in human malignancies, including Burkitt's lymphoma, MCL, and Hodgkin's lymphoma [21-25]. The repeated discovery of the involvement of NF-κB, STAT3 and PI3K in distinct forms of B-cell neoplasias underscores the importance of these signaling pathways in B-cell transformation.

Several findings support crosstalk among NF-κB, STAT3 and PI3K signaling in the iMyc^Eμ ^system. Inhibition of NF-κB abrogated constitutive STAT3 activity, inhibition of STAT3 reciprocally reduced constitutive NF-κB activity, and inhibition of PI3K suppressed activation of both NF-κB and STAT3 in iMyc^Eμ^-1 cells. When inhibitor combinations affecting NF-κB and STAT3 or either and PI3K were applied, additive suppression of proliferation was observed, indicating that the NF-κB and STAT3 pathways converge. The physical association between the active forms of NF-κB and STAT3 in iMyc^Eμ^-1 cells provides direct evidence for such crosstalk and convergence. Partial characterization of this complex revealed interactions between the NF-κB subunits p50, p65, and/or c-Rel, either directly or indirectly, with phosphorylated STAT3. The exact compositions of the complexes, and the ultimate functions of these interactions, are not yet defined. Although crosstalk among transcription factors is a common mode of gene regulation, and several studies have already reported physical and functional interactions between NF-κB and STAT3 in various cell types [42-46,64-66], to our knowledge, this is the first description of a physical association between NF-κB and STAT3 in neoplastic B cells. A recent study showed that constitutive STAT3 activity can maintain constitutive NF-κB activity in solid tumors [46], and our finding supports the possibility of a reciprocal activity of NF-κB and STAT3 in the maintenance of hematopoietic tumors.

We have explored the potential involvement of other pathways in the proliferation and survival of iMyc^Eμ ^-1 cells and on NF-κB and STAT3 signaling, but found that only the PI3K pathway was involved. It is very interesting that the Eμ-myc model of B-cell lymphoma, one of the earliest transgenic mice ever developed to still be widely used today [67], also showed a requirement for PI3K, but not mTOR or ERK, activity in mitogen-induced B-cell growth [68]. This suggests that the PI3K pathway may be a key modulator of Myc-driven B cell lymphomagenesis. Moreover, inhibition of PI3K abrogated STAT3 and NF-κB activity, and simultaneous inhibition of PI3K with NF-κB or STAT3 resulted in an additive growth inhibition, implying that PI3K functions upstream of NF-κB and STAT3 in iMyc^Eμ ^B cells. To follow up on how PI3K might be constitutively activated, we assessed the known causes of aberrant PI3K activity - loss or mutation of *Pten *[69-71] or mutation of *Pi3kca *[47,48,72,73] - but did not find these alterations in either LBLs or iMyc^Eμ^-1 cells. This finding is consistent with other studies indicating that neither PTEN nor *PI3KCA *is involved in B-cell malignancies [74,75]. The reason for constitutive activation of PI3K remains to be determined.

In keeping with our results, crosstalk among NF-κB, STAT3 and PI3K signaling is supported in the literature. Notable examples include AKT-mediated phosphorylation of IKK to activate NF-κB [76,77], IL-2-mediated induction of PI3K upstream of STAT3 activation in primary human T cells [78], and the physical interaction between the PI3K p85 subunit and STAT3 during STAT3 activation [79]. Furthermore, AKT, NF-κB and STAT3 signaling are required for the growth of lymphomas driven by the expression of Epstein-Barr Virus latent membrane protein 1 (EBV-LMP1) [50], and also for the survival of chronic lymphocytic leukemia (CLL) B cells [51]. Intriguingly, several recent reports describe a role for p300, an acetyltransferase, as a potential mediator of signaling crosstalk of NF-κB, STAT3 and PI3K/AKT. AKT-mediated phosphorylation of p300 dramatically increases its acetyltransferase activity and can increase acetylation and full transcriptional activation of p65 [80,81]. For STAT3, leukemia inhibitory factor (LIF)- or IL6-mediated activation of AKT can lead to phosphorylation of p300, and to subsequent acetylation and activation of STAT3 in 293T and Hep3B cells [65,82,83]. Also, acetylation of p65 by p300 is facilitated by STAT3 and can lead to enhanced nuclear localization of p65 [46]. Although proof the involvement of p300 in iMyc^Eμ ^B-cell neoplasia has not yet been demonstrated, p300 is a prime candidate to link the crosstalk of PI3K, NF-κB, and STAT3 signaling, and is of considerable interest for future studies.

To demonstrate that our results are not unique to iMyc^Eμ^-1 cells, we investigated whether similar signal transduction pathways were important for tumor maintenance in other mouse B-lymphoma lines (see additional files 5, 6, &7). Strikingly similar inhibitor sensitivity was seen in WEHI 231 and iMyc^Eμ^-1 cells. In fact, the sort of PI3K/NF-κB/STAT3 signaling crosstalk seen in iMyc^Eμ^-1 cells was also observed in WEHI 231 cells when we repeated many of the same experiments (see additional files 6 and 7). These findings argue that our results are not a peculiarity of iMyc^Eμ^-1 cells, and also make a strong case for the specificity of the small-molecule inhibitors used in our studies.

Premalignant B cells are difficult to obtain from humans, but mouse models, such as iMyc^Eμ ^are a ready source of these cells and can be used to elucidate the temporal regulation of molecular events in the course of lymphoma development. We found that NF-κB and STAT3 were already constitutively activated in splenic B cells of iMyc^Eμ ^mice months before overt tumors developed. The literature would suggest that this early activation of NF-kB and STAT3 is caused by an increase in IL6 and/or IL10 [4,44,49]. Our data are novel because they exclude the possibility of elevated IL6 or IL10 from either autocrine or paracrine sources in a pre-tumorigenic state. The reason for constitutive NF-kB and STAT3 activation remains unknown. Intriguingly, NF-κB and STAT3 are known to target Myc [34,38-41], but Myc protein was only slightly elevated during the premalignant stage in iMyc^Eμ ^mice. Some of our other results, however, are consistent with Myc as a target downstream of PI3K/NF-κB/STAT3 in tumors of the iMyc^Eμ ^system. Myc protein was highly elevated during malignancy, and inhibition of any one of the tested effectors of Myc transcription (PI3K, NF-κB or STAT3) resulted in a reduction of Myc protein. Moreover, a loss of Myc activity trailed the reduction of NF-κB and STAT3 activity after PI3K was inhibited in iMyc^Eμ^-1 cells. If Myc is upregulated by NF-κB and STAT3, perhaps this occurs at some point between the premalignant and malignant state in iMyc^Eμ ^B cells. Elucidating the nature of this apparent tumor progression event is ongoing in our laboratory, and will be the subject of a future manuscript.

## Conclusions

In summary, we provide evidence that PI3K, NF-κB and STAT3 are interconnected in iMyc^Eμ ^B cell lymphoma. Constitutive NF-κB and STAT3 activity are dependent upon one another, and both also depend on heightened PI3K activity. Signaling through each of these three molecules is required for tumor maintenance and Myc expression, and combined inhibition results in additive suppression of tumor growth. These findings, together with the fact that NF-κB and STAT3 physically associate with one another in the same complex, support the assertion that NF-κB and STAT3 converge downstream of PI3K in the development of iMyc^Eμ ^B-cell lymphoma. Our results underscrore the importance of further examination of crosstalk between NF-κB, STAT3 and PI3K in the development of Myc-driven B-cell neoplasia.

## Competing interests

The authors declare that they have no competing interests.

## Authors' contributions

SSH designed the study, performed most experiments, and wrote the article. DJS conducted proliferation assays, Co-IPs and the cytokine array experiments. HY performed Western blot analysis. VST contributed critical insights and edited the article. LP conducted FACS analysis. STC cultured cells and conducted proliferation assays. JK performed Western blot analysis for c-Myc. ESP contributed critical insights. SJ designed the study, evaluated results, and wrote and approved the article. All authors have read and approved the final manuscript.

## Supplementary Material

Additional file 1 **NF-κB and STAT3 are constitutively activated in splenic B cells of young iMyc^Eμ ^mice**. (A and B) EMSA showing an increase in DNA-binding for NF-κB (A) and STAT3 (B), respectively, beginning at one month of age and continuing through four months of age. Nuclear extracts from BL6-derived splenic B cells were used as a control (C). Click here for file

Additional file 2 **The Myc probe is specific**. (A) EMSA competition assay showing that 30-fold excess unlabeled probe successfully competes for Myc DNA-binding but a mutated probe does not. (B) Super-shift assay showing a loss of Myc DNA-binding that is specific for the Myc Ab but not for STAT3, p 50 or p 65 Abs. Click here for file

Additional file 3 **AKT is activated but PTEN message does not change in LBLs or iMyc^Eμ^-1 cells**. (A) Western blotting reveals the levels of total AKT protein and of phosphorylated AKT (S473 and T308) in the same samples, as in Figure 6*B *of Han et al. (2009). (B) Quantitation of PTEN mRNA, as shown in Figure 6*C *of Han et al. (2009). Click here for file

Additional file 4 **Inhibition of the PI3K pathway induces growth arrest and apoptosis of iMyc^Eμ^-1 cells**. (A) MTS/PMS anlysis reveals a dose-dependent decrease in cell proliferation after cells are treated with LY. Data were normalized to DMSO-treatment controls, and error bars represent the standard deviation from a representative experiment performed in triplicate. (B) DNA fragmentation was observed after treatment with LY, but not Rap, PD or SB, respectively. (C) Flow cytometry-based analyses of DNA content (PI; top row), as well as Annexin V (middle row) and cleaved caspase 3 (bottom row) levels, showing that treatment with PD, SB or Rap (open gray histogram) did not result in significant differences from untreated controls (filled black histogram). Treatments were for 24 hours and the inhibitor concentrations are indicated. Click here for file

Additional file 5 **Wehi 231cells are very similar to iMyc^Eμ^-1 cells with regard to NF-κB, STAT3 and PI3K signaling**. (A) EMSA showing constitutive activation of NF-κB, STAT3 and Myc in mouse BCL cell lines, as indicated. "C" denotes control BL6 splenic B cells. (B) Western blot comparing protein levels of AKT, P-AKT (S473 and T308), PTEN and α-tubulin in mouse BCL lines, as indicated. (C) MTS/PMS assay for proliferation of designated mouse BCL lines after treatment with vehicle control, LY, PD, SB, AEG, Rap, LC or WHI for 24 hours at the given concentrations. Data were normalized to DMSO controls, and error bars represent the standard deviation from a representative experiment performed in triplicate. Click here for file

Additional file 6 **In Wehi 231 cells, crosstalk among NF-κB, STAT3 and PI3K appears to regulate Myc**. (A) EMSA revealing that binding of NF-κB, STAT3 and Myc to DNA is sensitive to inhibition of PI3K (LY), NF-κB (LC), STAT3 (WHI) and JNK (AEG), but not PD, SB or Rap. (B) NF-κB, STAT3 and Myc DNA-binding activity is reduced in a time-dependent manner after PI3K is inhibited with LY. (C and D) EMSA super-shift assays performed with STAT3-specific probes and NF-κB-specific Abs (C) or NF-κB-specific probes and STAT3-specific Abs (D), respectively. Abs were specific for subunits of NF-κB, Tyr-705 phosphorylated STAT3 (P-STAT3) and total STAT3 as indicated. SP1 and Myc Abs were used as negative controls. (E) Co-IP and Western blot showing co-immunoprecipitation of NF-κB p50 and P-STAT3. Abs used for immunoprecipitations (IP) and Western blotting (WB) are designated. The incubation time with small-molecule inhibitors was 24 hours unless otherwise noted. Click here for file

Additional file 7 **Co-treatment with small-molecule inhibitors of NF-κB, STAT3 and/or PI3K additively inhibits proliferation of Wehi 231 cells**. MTS/PMS cell proliferation assay after cell treatment with low doses of LY, LC and WHI, either in isolation or in various combinations, for 24 hours. Box at bottom gives the average percent (%) growth inhibition. Data were normalized to DMSO controls, and error bars represent the standard deviation from a representative experiment performed in triplicate. Click here for file
