# Supplementary figures and images for: NF-κB/STAT3/PI3K signaling crosstalk in iMycEμ B lymphoma
Source: Mol Cancer. 2010 Apr 30;9:97. doi: 10.1186/1476-4598-9-97 (PMC2876994; doi:10.1186/1476-4598-9-97)

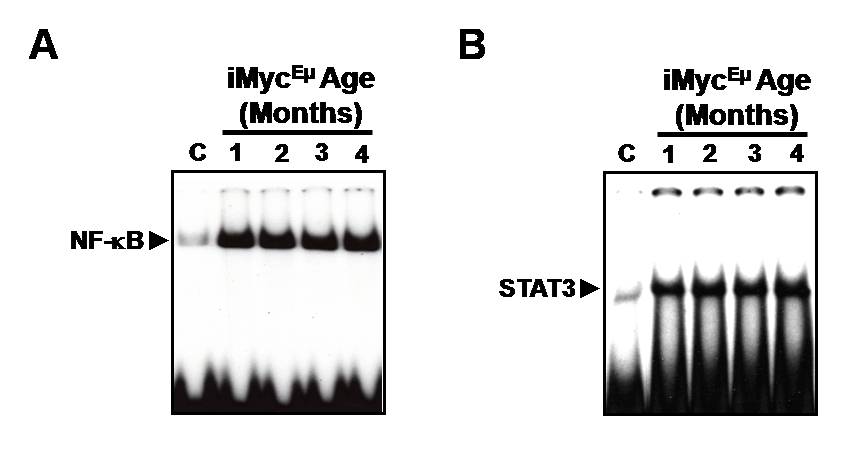

Supplement: Additional file 1 — NF-κB and STAT3 are constitutively activated in splenic B cells of young iMycEμ mice. (A and B) EMSA showing an increase in DNA-binding for NF-κB (A) and STAT3 (B), respectively, beginning at one month of age and continuing through four months of age. Nuclear extracts from BL6-derived splenic B cells were used as a control (C). [file 1476-4598-9-97-S1.JPEG]

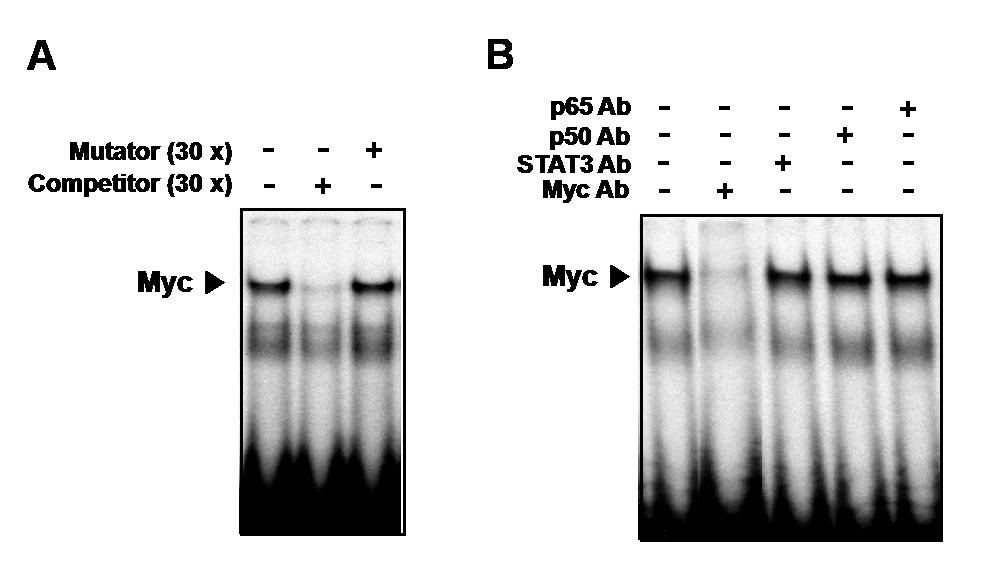

Supplement: Additional file 2 — The Myc probe is specific. (A) EMSA competition assay showing that 30-fold excess unlabeled probe successfully competes for Myc DNA-binding but a mutated probe does not. (B) Super-shift assay showing a loss of Myc DNA-binding that is specific for the Myc Ab but not for STAT3, p 50 or p 65 Abs. [file 1476-4598-9-97-S2.JPEG]

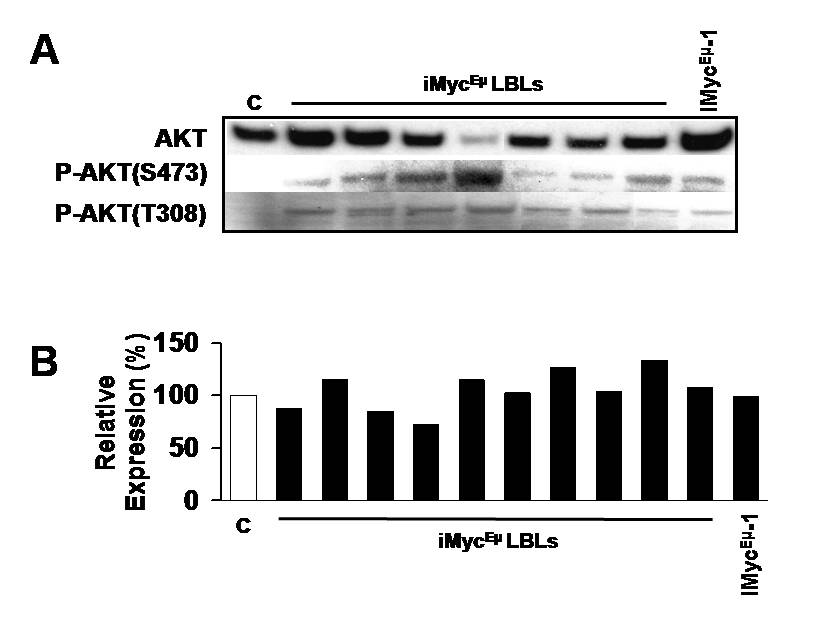

Supplement: Additional file 3 — AKT is activated but PTEN message does not change in LBLs or iMycEμ-1 cells. (A) Western blotting reveals the levels of total AKT protein and of phosphorylated AKT (S473 and T308) in the same samples, as in Figure 6B of Han et al. (2009). (B) Quantitation of PTEN mRNA, as shown in Figure 6C of Han et al. (2009). [file 1476-4598-9-97-S3.JPEG]

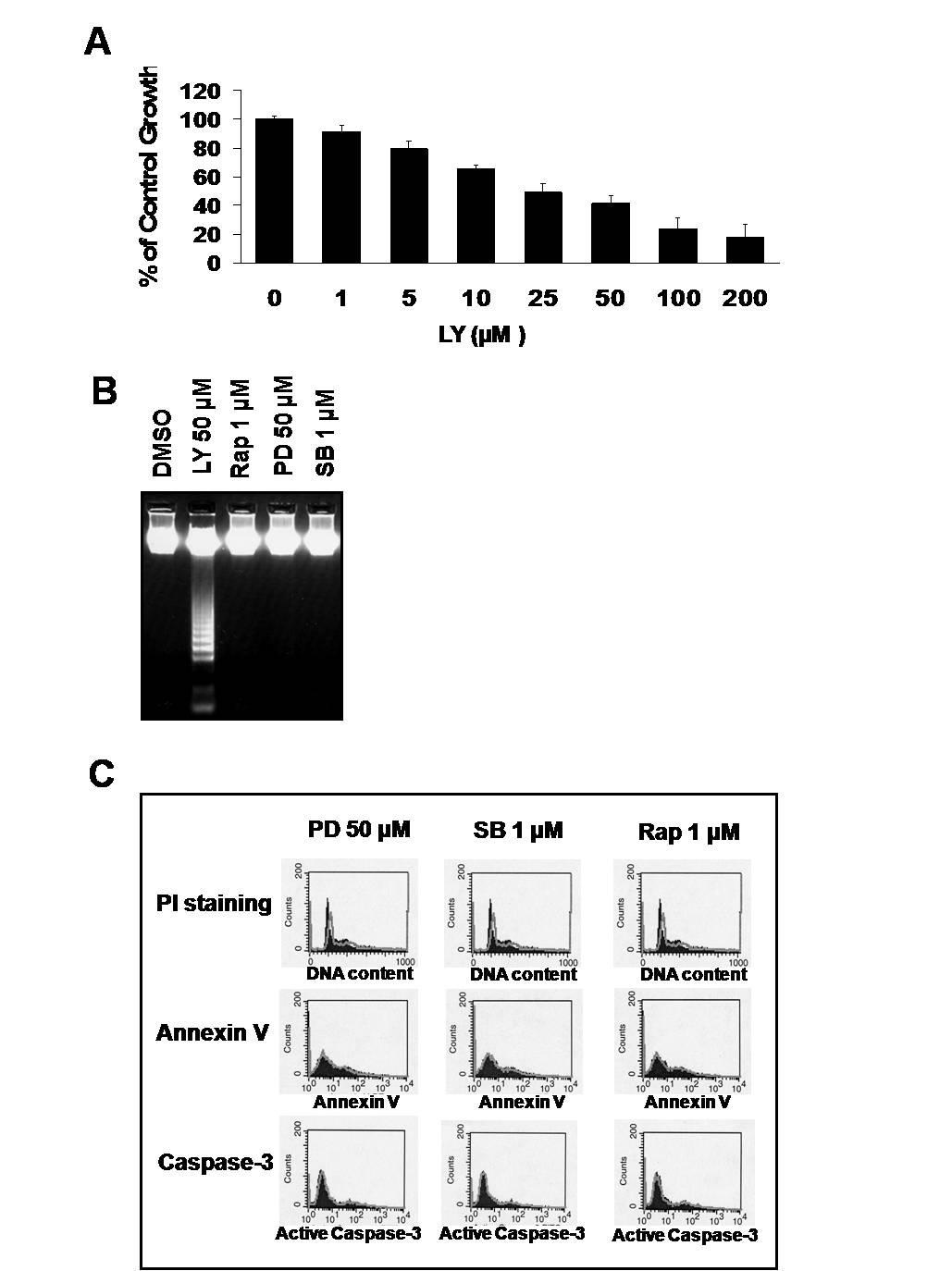

Supplement: Additional file 4 — Inhibition of the PI3K pathway induces growth arrest and apoptosis of iMycEμ-1 cells. (A) MTS/PMS anlysis reveals a dose-dependent decrease in cell proliferation after cells are treated with LY. Data were normalized to DMSO-treatment controls, and error bars represent the standard deviation from a representative experiment performed in triplicate. (B) DNA fragmentation was observed after treatment with LY, but not Rap, PD or SB, respectively. (C) Flow cytometry-based analyses of DNA content (PI; top row), as well as Annexin V (middle row) and cleaved caspase 3 (bottom row) levels, showing that treatment with PD, SB or Rap (open gray histogram) did not result in significant differences from untreated controls (filled black histogram). Treatments were for 24 hours and the inhibitor concentrations are indicated. [file 1476-4598-9-97-S4.JPEG]

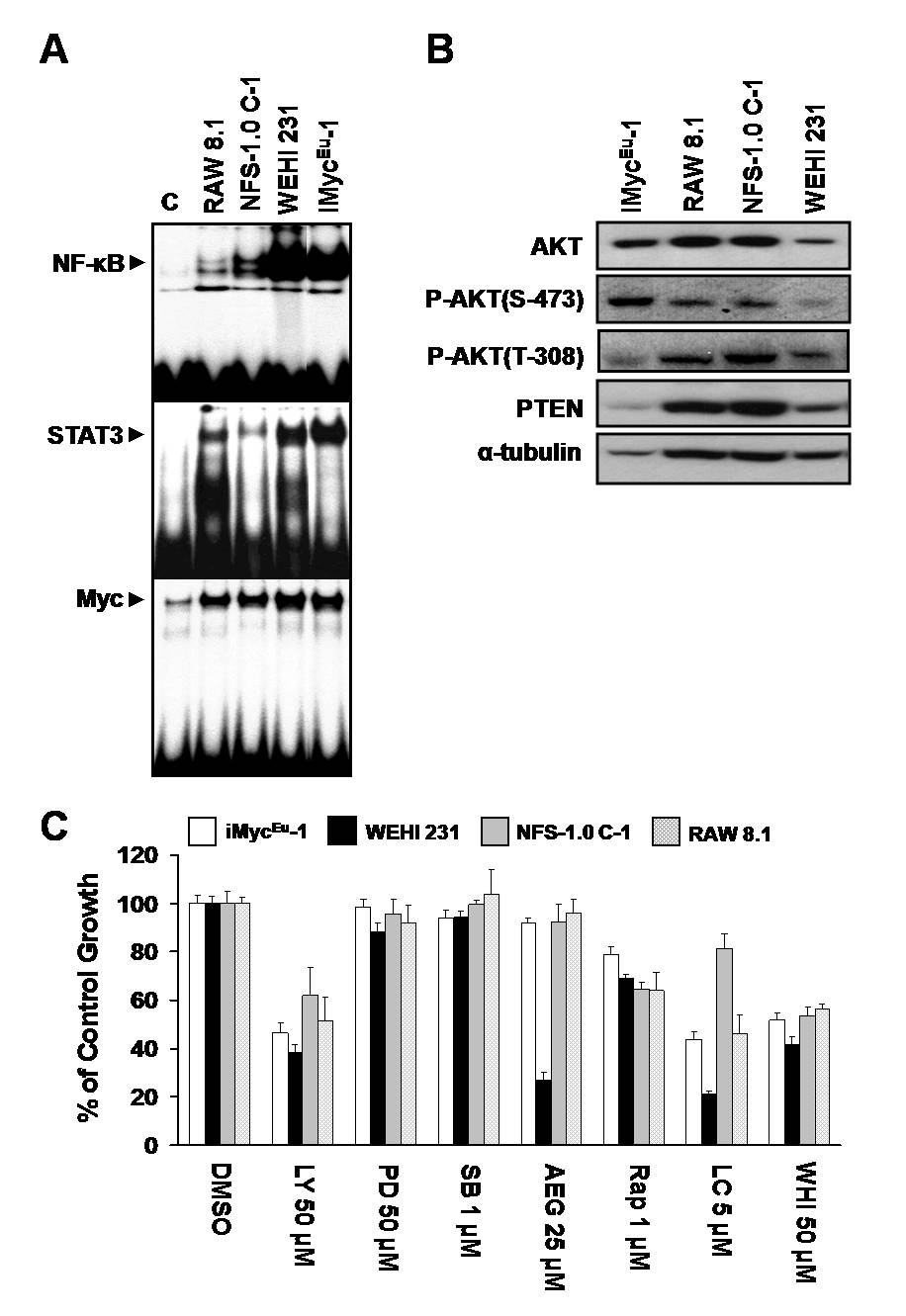

Supplement: Additional file 5 — Wehi 231cells are very similar to iMycEμ-1 cells with regard to NF-κB, STAT3 and PI3K signaling. (A) EMSA showing constitutive activation of NF-κB, STAT3 and Myc in mouse BCL cell lines, as indicated. "C" denotes control BL6 splenic B cells. (B) Western blot comparing protein levels of AKT, P-AKT (S473 and T308), PTEN and α-tubulin in mouse BCL lines, as indicated. (C) MTS/PMS assay for proliferation of designated mouse BCL lines after treatment with vehicle control, LY, PD, SB, AEG, Rap, LC or WHI for 24 hours at the given concentrations. Data were normalized to DMSO controls, and error bars represent the standard deviation from a representative experiment performed in triplicate. [file 1476-4598-9-97-S5.JPEG]

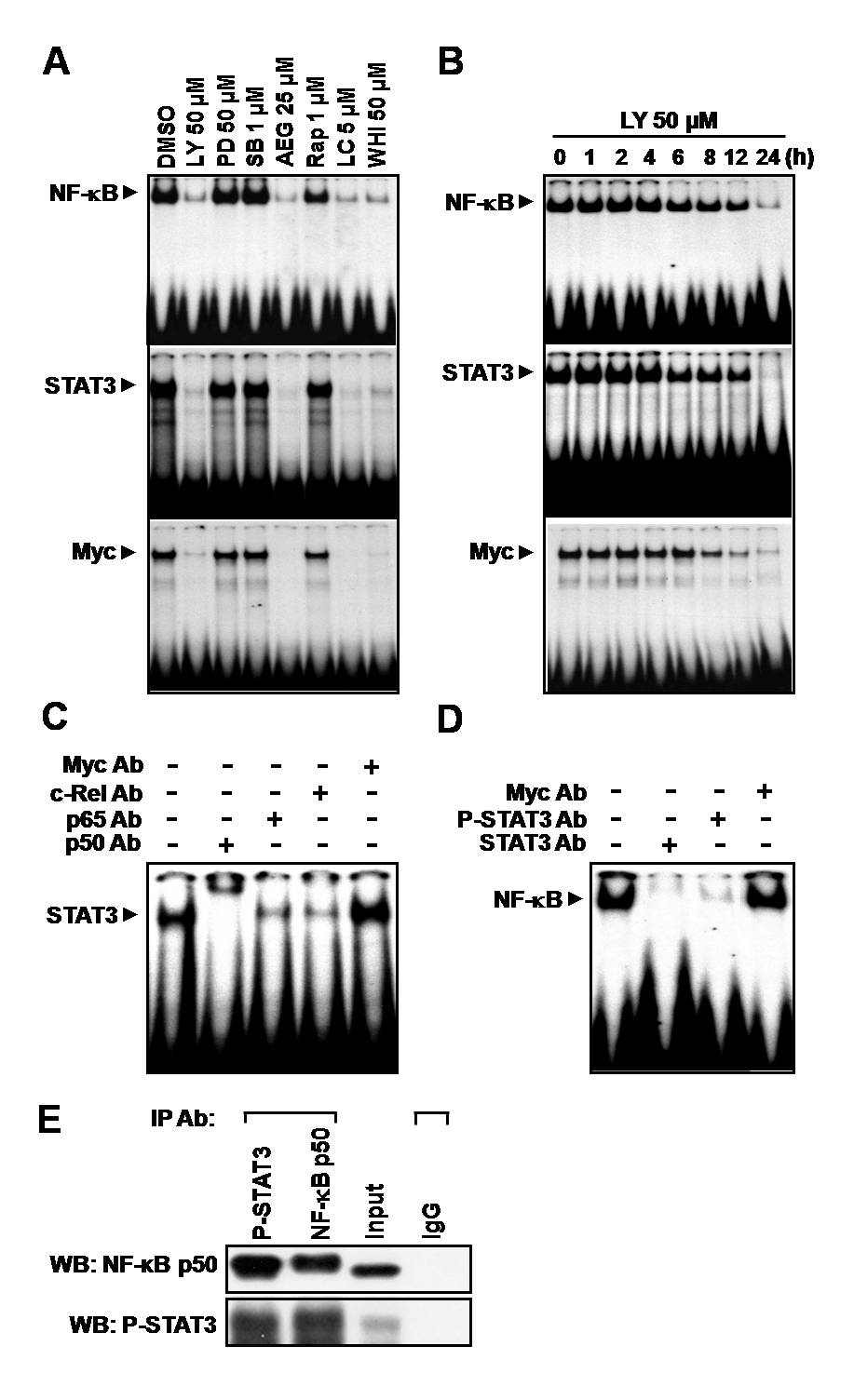

Supplement: Additional file 6 — In Wehi 231 cells, crosstalk among NF-κB, STAT3 and PI3K appears to regulate Myc. (A) EMSA revealing that binding of NF-κB, STAT3 and Myc to DNA is sensitive to inhibition of PI3K (LY), NF-κB (LC), STAT3 (WHI) and JNK (AEG), but not PD, SB or Rap. (B) NF-κB, STAT3 and Myc DNA-binding activity is reduced in a time-dependent manner after PI3K is inhibited with LY. (C and D) EMSA super-shift assays performed with STAT3-specific probes and NF-κB-specific Abs (C) or NF-κB-specific probes and STAT3-specific Abs (D), respectively. Abs were specific for subunits of NF-κB, Tyr-705 phosphorylated STAT3 (P-STAT3) and total STAT3 as indicated. SP1 and Myc Abs were used as negative controls. (E) Co-IP and Western blot showing co-immunoprecipitation of NF-κB p50 and P-STAT3. Abs used for immunoprecipitations (IP) and Western blotting (WB) are designated. The incubation time with small-molecule inhibitors was 24 hours unless otherwise noted. [file 1476-4598-9-97-S6.JPEG]

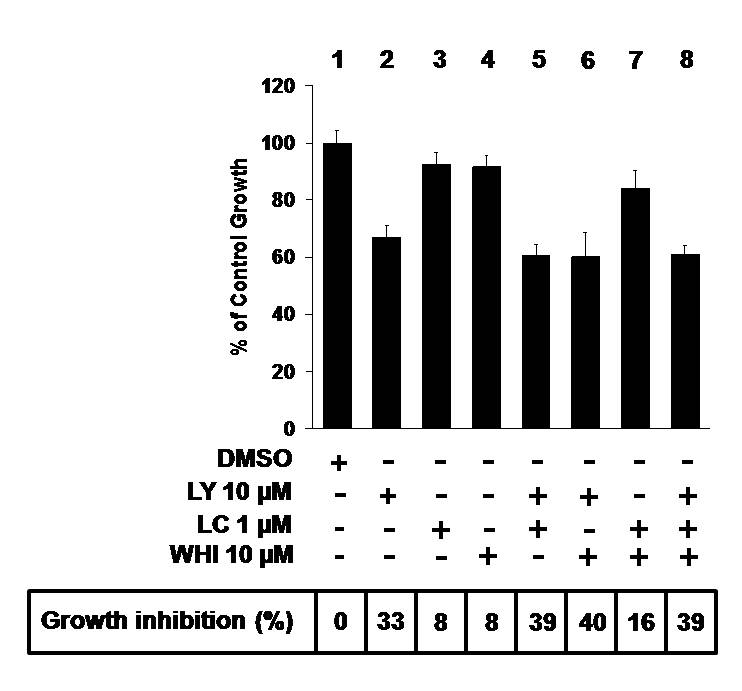

Supplement: Additional file 7 — Co-treatment with small-molecule inhibitors of NF-κB, STAT3 and/or PI3K additively inhibits proliferation of Wehi 231 cells. MTS/PMS cell proliferation assay after cell treatment with low doses of LY, LC and WHI, either in isolation or in various combinations, for 24 hours. Box at bottom gives the average percent (%) growth inhibition. Data were normalized to DMSO controls, and error bars represent the standard deviation from a representative experiment performed in triplicate. [file 1476-4598-9-97-S7.JPEG]
